# Associations between common sleep disturbances and cardiovascular risk in patients with obstructive sleep apnea: A large-scale cross-sectional study

**DOI:** 10.3389/fcvm.2022.1034785

**Published:** 2022-10-31

**Authors:** Xiaoman Zhang, Weijun Huang, Huajun Xu, Xinyi Li, Fan Wang, Kejia Wu, Chenyang Li, Yupu Liu, Jianyin Zou, Huaming Zhu, Hongliang Yi, Jian Guan, Di Qian, Shankai Yin

**Affiliations:** ^1^Department of Otolaryngology Head and Neck Surgery and Shanghai Key Laboratory of Sleep Disordered Breathing, Shanghai Sixth People’s Hospital Affiliated to Shanghai Jiao Tong University School of Medicine, Otolaryngology Institute of Shanghai Jiao Tong University, Shanghai, China; ^2^Department of Otolaryngology, People’s Hospital of Longhua, Shenzhen, China

**Keywords:** obstructive sleep apnea, cardiovascular disease, slow-wave sleep, excessive daytime sleepiness, sleep duration

## Abstract

**Objectives:**

Studies have shown that obstructive sleep apnea (OSA) is inextricably linked with cardiovascular diseases (CVD). However, the roles of certain common sleep disturbances, such as low slow-wave sleep, excessive daytime sleepiness and short sleep duration, in the pathogenesis and progression of CVD in patients with OSA have not been determined. Therefore, we conducted a large cross-sectional study to explore the effect of low slow-wave sleep, excessive daytime sleepiness and short sleep duration on the risk of CVD in patients with OSA.

**Methods:**

Subjects were consecutively enrolled to participate in the sleep center of Shanghai Jiao Tong University Affiliated Sixth People’s Hospital. All OSA patients were diagnosed by standard polysomnography, while controls were all simple snorers. A total of 4,475 participants were strictly recruited. The Framingham Risk Score were employed to assess the 10-year risk of CVD, and logistic regression was used to measure the association between sleep disturbances and the moderate-to-high CVD risk.

**Results:**

In the whole cohort, OSA, excessive daytime sleepiness, and low slow-wave sleep were all risk factors for the moderate-to-high 10-year CVD risk (odds ratio [OR] = 3.012, 95% confidence interval [CI] 2.418–3.751; OR = 1.407, 95% CI: 1.228–1.613, and OR = 0.973,95% CI: 0.967–0.980), but sleep duration did not contribute significantly to that risk. Whether in patients with OSA and controls, low SWS (<12.8%) could increase the risk of CVD. Subjective excessive daytime sleepiness would significantly increase the risk of CVD only in patients with severe OSA.

**Conclusion:**

It is important to pay more attention to the impact of sleep on cardiovascular health. Patients with sleep disturbances should adopt a healthy lifestyle and undergo regular follow-up of cardiovascular indicators to prevent cardiovascular complications.

**Trial registration:**

[http://www.chictr.org.cn/showproj.aspx?proj=43057], identifier [ChiCTr1900025714].

## Highlights

- What is already known on this topic: The roles of certain common sleep disturbances, such as low slow-wave sleep, excessive daytime sleepiness, and short sleep duration, in the pathogenesis and progression of cardiovascular diseases have not been determined in patients with obstructive sleep apnea.
- What this study adds: This is the first study to investigate the relationship between objective and subjective sleep parameters and cardiovascular diseases comprehensively.
- How this study might affect research, practice, or policy: In control subjects and patients with OSA, low SWS increases the risk of CVD. Only in patients with severe OSA, subjective EDS significantly increases the risk of CVD. Sleep duration seems to play a minor role in the CVD risk. These findings will give reference for further clinical trials.

## Introduction

Cardiovascular disease (CVD) is the leading cause of death in China, accounting for more than 40% of all deaths (1, 2). Much effort has been devoted to CVD prevention and control over the past few decades. However, current strategies are not effective, possibly because some important risk factors for CVD may be unknown. Sleep may be significant in terms of both the pathogenesis and progression of CVD. Sleep is an important regulator of cardiovascular function under both physiological and pathological conditions. Under physiological conditions, sleep plays pivotal roles in the autonomic nervous system, systemic hemodynamics, cardiac and endothelial function (3). A cross-sectional study found strong associations between primary sleep disturbances (abnormal sleep duration, shift work, and sleep apnea) and CVD (4). In addition, screenings of persons with stressful jobs revealed that the prevalence of self-reported psychosomatic diseases (diabetes and CVD) was higher in people with sleep disorders than those without (5, 6).

Obstructive sleep apnea (OSA) refers to a series of pathophysiological changes (including sleep apnea and sleep structure disorders) caused by frequent collapse and obstruction of the upper airway during sleep (7). OSA affects 936 million adults (aged 30–69 years) worldwide (8). Increasing evidence has shown that OSA is associated with a series of cardio- and cerebro-vascular comorbidities, including hypertension, coronary artery disease, stroke, and heart failure (9–12). OSA may contribute to the development of metabolic and cardiovascular events by stimulating the sympathetic nervous system, changing the mechanical and hemodynamic parameters, and inducing oxidative stress (13, 14). Slow-wave sleep (SWS), also termed N3 sleep, is characterized by ≥20% slow waves featuring a low frequency band (0.5–4.5 Hz) (15). Electroencephalogram (EEG) can be used to divide sleep into non-rapid eye movement (NREM) and rapid eye movement (REM) phases. NREM can be further divided into three stages (N1, N2, and N3) as sleep progresses from shallow to deep. SWS accounts for 10∼25% of all sleep in young healthy adults (16). Genetic factors and environmental factors such as alcohol use, drugs, and sleep disorders diseases (15, 17, 18) affect the proportion of SWS; such phase features a gradual decrease in sympathetic activity accompanied by parasympathetic innervation, a downward reset of the arterial baroreceptor reflex, and decreases in the heart rate, cardiac output, and blood pressure. A reduction in SWS associated with OSA and its related frequent arousals excessively activates the sympathetic nerves, increasing the risk of cardiovascular and metabolic diseases (19). Excessive daytime sleepiness (EDS), which can be defined by the Epworth sleepiness scale (ESS) score > 10, is one of the principal symptoms of OSA. EDS refers to the difficulties in concentration during work, creating major public health risks, including motor vehicle accidents and medical errors by healthcare workers. However, EDS is not entirely caused by health consequences, being also related to working hours, work pressures, and family responsibilities (20). Current studies have reported that EDS is associated with the increased risks of CVD and coronary heart disease, but only slightly (or not at all) with the risk of stroke (21, 22). Short sleep duration (SSD) may be caused by sleep disorders, living habits, or the sleep environment (23). The US National Sleep Foundation states that sleep durations < 6 h for adults 26–64 years of age and 5–6 h for older adults ≥ 65 years of age are insufficient (24). Sleep that is too short or too long may increase the risks of hypertension and coronary artery disease, suggesting that the impact of sleep duration on cardiovascular events might be U-shaped (25, 26). However, most studies on sleep duration used self-reporting questionnaires to evaluate total sleep time; bias might be in play. Also, it is unknown whether the association between sleep duration and cardiovascular events is related to OSA.

In total, few studies have comprehensively explored whether aforementioned common sleep disturbances (low SWS, EDS, and SSD) affect the risk of CVD in patients with OSA revealed by standard polysomnography (PSG). We thus performed a large-scale cross-sectional study contribution to the field of the pathogenesis of OSA-associated CVD, and formulating prevention, treatment, and management strategies for OSA-associated CVD.

## Materials and methods

### Subject population

Subjects were consecutively enrolled to participate in the sleep center of Shanghai Jiao Tong University Affiliated Sixth People’s Hospital. This study was performed in accordance with the Declaration of Helsinki and was approved by the Ethics Committee of Shanghai Jiao Tong University Affiliated Sixth People’s Hospital (trial registration No: ChiCTr1900025714). Informed consent was obtained from all participants. All participants came to the hospital to seek medical advice because of snoring. They were all adults (age ≥ 18 years) and underwent standard PSG. All OSA patients were diagnosed with apnea hypopnea index (AHI) ≥ 5event/h, while controls with apnea hypopnea index (AHI) < 5event/h. The control group were all simple snorers and voluntarily included in the study. The screening and enrollment process of participants was shown in the Supplementary Figure 1. The following were the exclusion criteria of the study: (1) absence of important data records; (2) age < 18 years; (3) pre-existing of CVD, including heart failure and coronary ischemia; (4) previous interventions for OSA, such as upper-airway surgery and/or continuous positive airway pressure (CPAP); and (5) severe comorbidities, including renal failure and malignant tumors. Finally, 4475 subjects were included.

### Patient and public involvement

Patients were not involved in the design, recruitment to or conduct of the studies included in this analysis.

### Data collection

#### Sleep evaluation

All enrolled participants completed the eight-item Epworth Sleepiness Scale (ESS) to assess subjective daytime sleepiness (27). An ESS score > 10 was defined as excessive daytime sleepiness (EDS). Standard PSG (Alice 4 or 5; Respironics, Pittsburgh, PA, USA) was used to evaluate objective sleep status according to the American Academy of Sleep Medicine (AASM) 2007 criteria (28). Each full-night PSG session yielded an electroencephalogram (EEG), an electrocardiogram (ECG), a bilateral electrooculogram (EOG), a genioglossus electromyogram, and information on thoracic-abdominal movement, nose and mouth airflows, and finger pulse oxygen saturation, etc.

#### Clinical and biochemical tests

Prior to PSG, all participants were measured height, weight, waist circumference (WC), hip circumference (HC), and neck circumference (NC). Systolic blood pressure (SBP) and diastolic blood pressure (DBP) were measured using a standard mercury sphygmomanometer after at least 15 min of rest. All of these parameters were measured twice and averaged. At 7:00 AM on the morning after PSG, fasting venous blood was collected to construct fasting lipid profile (total cholesterol [TC], high-density-lipoprotein C [HDL-c], and low-density-lipoprotein C [LDL-c]). The diagnosis of diabetes and hypertension was self-reported or based on the prior diagnosis and the use of relevant drugs. Hyperlipidemia was determined on the basis of the medical history and the lipid index measurements.

#### Definitions and calculations

The body mass index (BMI) was calculated as weight (kg) divided by the square of height (m^2^). The waist-to-hip ratio (WHR) was defined as WC/HC. Homeostasis model assessment of insulin resistance (HOMA-IR) was counted as fasting insulin (μU/mL) *fasting glucose (mmol/L)/22.5 (29). HOMA-IR > 2.7 was defined as IR in this study (30).

The parameters derived from full-night PSG included apnea hypopnea index (AHI), total sleep time (TST), the percentage of each sleep stage (N1, N2, N3, and REM), sleep efficiency (SE), lowest oxygen saturation (LSpO2), oxygen desaturation index (ODI), mean oxygen saturation, and microarousal index (MAI). Sleep variables were defined in line with the American Academy of Sleep Medicine (28). Apnea was defined as an ≥90% reduction in airflow for at least 10 s; hypopnea as ≥50% reduction in airflow for ≥10 s accompanied by ≥3% decrease in oxygen saturation or arousal. AHI referred to the number of apnea and hypopnea per hour. Subjects with AHI < 5 served as controls, while patients with AHI ≥ 5 were considered as OSA group. According to AHI, patients with OSA were classified into three groups: mild (5 ≤ AHI < 15), moderate (15 ≤ AHI < 30), and severe (AHI ≥ 30). Sleep duration was evaluated according to the TST and was divided into five categories: ≥8, 7–8, 6–7, 5–6, and <5 h. A sleep duration of 7–8 h served as the reference. SWS was the percentage of the N3 phase over the TST and was divided into four quartiles (for the whole cohort: ≥20.1, 12.8–20.1, 5.9–12.8, and <5.9%). The highest quartile served as the reference.

The 10-year CVD risk was assessed using the Framingham Risk Score (FRS), which could predict the risk of future coronary heart disease events and aid preventative management. The FRS was evaluated by seven factors including age, gender, smoking and diabetes status, the HDL and TC levels, and the SBP (31). Participants with risk < 10% were considered low risk, 10∼20% at moderate risk, and >20% at high risk. We divided subjects into those at low risk (<10%) and moderate-to-high risk (≥10%).

### Statistical analyses

All data were processed through IBM SPSS Statistics ver. 26.0. Categorical variables were described by numbers and percentage. We used Kolmogorov–Smirnov test to divide continuous variables into those that were normally distributed data and skewed. The former data were expressed as mean ± standard deviation and the latter as median and interquartile range. The chi-squared test and non-parametric tests were used (as appropriate) to analyze differences between groups. Logistic regression was employed to identify factors contributing to a moderate-to-high risk of CVD; risk factors with *P*-values < 0.15 on univariate analysis were included in multivariate analysis. Odds ratios (ORs) and 95% confidence intervals (CIs) were calculated via logistic regression. Two-sided *P*-values < 0.05 were considered statistically significant.

## Results

### Baseline characteristics

The study totally included 4,475 participants, of whom 2,934 were at low risk of CVD and 1,541 at moderate-to-high risk. The median ages of these two groups were 36 (31–42) and 53 (46–60) years old (*P* < 0.001). All of the NC, WC, HC, WHR, BMI, SBP, DBP, lipid profiles, fasting glucose and insulin levels were higher in the latter group. In general, those at moderate-to-high risk of CVD evidenced larger anthropometric parameters and more severe metabolic characteristics. Compared to the low-risk group, those at moderate-to-high risk of CVD exhibited significant differences in sleep parameters, such as higher AHI, ODI, MAI, and lower mean or lowest SpO2. Participants at moderate-to-high risk of CVD had higher proportion of OSA (*n* = 1430, 92.8%) and EDS (*n* = 659, 42.8%) than those at low risk (*n* = 2222, 75.7%; *n* = 871, 29.7%). Although there was no significant difference in the TST between these two groups, the sleep structure of patients at moderate-to-high risk of CVD differed from that of the other. Compared to the low-risk group, the proportion of N1 phase increased whereas that of N3 decreased in the moderate-to-high risk group. The detailed demographic, clinical, and sleep characteristics of this sample was listed in Table 1.

**TABLE 1:** The demographic, clinical, and sleep characteristics of the overall participants stratified by the FRS.

Characteristics	Whole cohort *n* = 4475		
	Low CVD risk (FRS < 10%) *n* = 2934	Moderate-to-high CVD risk (FRS ≥ 10%) *n* = 1541	*P*
** Demographic and clinical characteristics**			
Men *n* (%)	2273 (77.5%)	1327 (86.1%)	<0.001
Age (y)	36 (31–42)	53 (46–60)	<0.001
Neck circumference (cm)	39.00 (36.450–41.00)	40 (38–42)	<0.001
Waist circumference (cm)	93 (86–100)	98 (92–104)	<0.001
Hip circumference (cm)	100 (96–105)	102 (98–106)	<0.001
Waist/Hip	0.931 (0.890–0.969)	0.962 (0.928–1.000)	<0.001
BMI (kg/m2)	25.949 (23.525–28.374)	26.754 (24.859–29.309)	<0.001
Hypertension	425 (14.5%)	887 (57.6%)	<0.001
SBP (mmHg)	120 (118-127)	130 (120–140)	<0.001
DBP (mmHg)	80 (75–81)	81 (80–90)	<0.001
Diabetes *n* (%)	40 (1.4%)	206 (13.4%)	<0.001
Hyperlipidemia *n* (%)	367 (12.5%)	497 (32.3%)	<0.001
Smoker *n* (%)	855 (29.1%)	927 (60.2%)	<0.001
Alcohol use *n* (%)	1310 (44.6%)	781 (50.7%)	<0.001
Fasting glucose (mmol/l)	5.130 (4.830–5.493)	5.480 (5.070–6.155)	<0.001
Fasting insulin (μU/ml)	10.150 (6.870–15.190)	11.210 (7.600–16.100)	<0.001
HOMA-IR	2.321 (1.529–3.616)	2.780 (1.816–4.225)	<0.001
Cholesterol (mmol/l)	4.610 (4.050–5.190)	4.870 (4.230–5.545)	<0.001
Triglyceride (mmol/l)	1.470 (1.010–2.150)	1.78 (1.265–2.595)	<0.001
HDL (mmol/l)	1.050 (0.910–1.220)	1.010 (0.880–1.130)	<0.001
LDL-c (mmol/l)	2.890 (2.400–3.380)	3.030 (2.460–3.610)	<0.001
ESS	7 (3–12)	9 (5–14)	<0.001
ESS > 10 *n* (%)	871 (29.7%)	659 (42.8%)	<0.001
** Polysomnography**			
TST (min)	411.602 (358.696–450.000)	414.894 (357.619–452.094)	0.349
Sleep efficiency	0.956 (0.881–0.989)	0.946 (0.867–0.986)	0.011
S1/TST (%)	14.300 (8.000–22.825)	17.200 (8.900–27.200)	<0.001
S2/TST (%)	53.500 (43.775–61.800)	53.500 (41.900–62.200)	0.676
S3/TST (%)	14.200 (7.300–21.425)	10.200 (3.300–17.350)	<0.001
REM/TST (%)	10.800 (6.400–15.000)	9.900 (5.600–14.200)	<0.001
AHI	21.350 (5.300–53.300)	42.300 (20.250–60.250)	<0.001
OSA *n* (%)	2222 (75.7%)	1430 (92.8%)	<0.001
Lowest SaO2	83 (72–90)	77 (67–85)	<0.001
Mean SaO2	95 (93.2–96)	94 (92–95)	<0.001
ODI	21.500 (5.300–53.825)	42.800 (20.450–61.000)	<0.001
MAI	20.600 (12.300–36.325)	26.600 (15.00–44.200)	<0.001

The data are presented as means and standard deviation; skewed data are presented as the median (IQR), and categorical data as the number (percentage). Differences in the baseline characteristics among the two groups were examined using chi-square test or non-parametric tests. BMI, body mass index; SBP, systolic blood pressure; DBP, diastolic blood pressure; HOMA-IR, homeostasis model assessment of insulin resistance; HDL-C, high-density lipoprotein cholesterol; LDL-C, low-density lipoprotein; ESS, Epworth sleepiness scale; TST, total sleep time; REM: rapid eye movement; AHI, apnea hypopnea index; OSA, obstructive sleep apnea; SaO2, oxygen saturation; ODI, oxygen desaturation index; MAI, microarousal index; FRS, 10-year Framingham CVD risk score.

### Factors contributing to the moderate-to-high 10-year cardiovascular diseases risk

In order to explore the risk factors contributing to the moderate-to-high 10-year CVD risk, we first selected sleep data (OSA, SWS, EDS, and sleep duration) and then factors (BMI ≥ 25, TG, LDL-c, HOMA-IR > 2.7, and hyperlipidemia) that might affect the CVD risk on the basis of the previous literature; we performed univariate and multivariate logistic regression (Table 2). In this model, TG, LDL-c, the presence of hyperlipidemia, OSA, ESS > 10, and SWS were all the risk factors for CVD (OR = 1.080, 95% CI: 1.037–1.125; OR = 1.157, 95% CI: 1.069–1.253; OR = 2.811, 95% CI: 2.396–3.299; OR = 3.012, 95% CI: 2.418–3.751; OR = 1.407, 95% CI: 1.228–1.613, and OR = 0.973,95% CI: 0.967–0.980, respectively) after adjustment. However, no significant association was apparent between sleep duration and the moderate-to-high 10-year CVD risk. In addition, multinomial logistic analysis was also performed to evaluate the association between risk factors and 10-year risk for cardiovascular disorders (according to FRS scoring into three separate groups) (Supplementary Table 1). The results were consistent with those in Table 2. TG, LDL-c, HOMA-IR, the presence of hyperlipidemia, OSA, ESS > 10, and SWS were all the risk factors for CVD risk. However, no significant association was apparent between sleep duration and the moderate-to-high 10-year CVD risk.

**TABLE 2:** Logistic regression analysis of factors associated with a moderate-to-high Framingham CVD risk in overall participants.

Characteristics	Univariate analysis			Multivariate analysis		
	OR 95%CI P			OR 95%CI P		
BMI ≥ 25	0.891	0.784–1.013	0.077			
TG	1.174	1.127–1.233	<0.001	1.080	1.037–1.125	<0.001
LDL-c	1.244	1.155–1.340	<0.001	1.157	1.069–1.253	<0.001
HOMA-IR > 2.7	1.619	1.429–1.833	<0.001			
Hyperlipidemia	3.330	2.858–3.880	<0.001	2.811	2.396–3.299	<0.001
OSA	4.128	3.344–5.097	<0.001	3.012	2.418–3.751	<0.001
TST	1.000	0.999–1.001	0.990			
ESS > 10	1.770	1.557–2.012	<0.001	1.407	1.228–1.613	<0.001
SWS	0.969	0.963–0.975	<0.001	0.973	0.967–0.980	<0.001

CVD, cardiovascular diseases; OR, odds ratio; CI, confidence interval; BMI, body mass index; TG, triglyceride; LDL-C, low-density lipoprotein; HOMA-IR, homeostasis model assessment for insulin resistance; OSA, obstructive sleep apnea; TST, total sleep time; ESS, Epworth sleepiness scale; SWS, slow-wave sleep.

### Subgroup analyses of associations between the 10-year cardiovascular diseases risk and obstructive sleep apnea, short sleep duration, excessive daytime sleepiness, and low slow-wave sleep

To evaluate further the dose-dependent associations of OSA, SWS, EDS, and SSD with the CVD risk, we performed additional subgroup analyses (Table 3). Model 1 featured univariate logistic analysis; Model 2 was a multivariate analysis after adjusting for sex, BMI and alcohol use; Model 3 was Model 2 with additional adjustment for the fasting glucose, LDL-c, and hyperlipidemia; and Model 4 was Model 3 with adjustment for sleep variables (MAI, SE, TST, ESS, SWS, and AHI). Specifically, when assessing the relationship between SWS and CVD risk, MAI, SE, TST, ESS, and AHI were added for adjustment; when assessing the relationship between EDS and CVD risk, MAI, SE, TST, SWS, and AHI were added for adjustment; when assessing the relationship between TST and CVD risk, MAI, SE, ESS, SWS, and AHI were added for adjustment, when assessing the relationship between OSA and CVD risk, MAI, SE, TST, ESS, and SWS were added for adjustment. In the whole cohort, OSA was independently associated with the moderate-to-high CVD risk irrespective of OSA severity, whether adjusted or not for metabolic or sleep factors. With the increase of OSA severity, the relationship between OSA and moderate-to-high CVD risk was strengthened, that is, the risk of CVD increased with the trend of OSA severity (OR = 1.551, 95% CI: 1.172–2.052; OR = 3.041, 95% CI: 2.338–3.955; OR = 3.365, 95% CI: 2.626–4.310). In addition, low SWS was significantly associated with the moderate-to-high CVD risk and the association became stronger as SWS decreased (OR = 1.243, 95% CI: 1.019–1.516; OR = 1.597, 95% CI: 1.315–1.940; OR = 2.139, 95% CI: 1.763–2.595) compared to the highest quartile. We also found a strong association between EDS and the moderate-to-high CVD risk. However, after stratification, there was no significant association between sleep duration and the CVD risk.

**TABLE 3:** Association of OSA, SWS, EDS, SSD and the moderate-to-high Framingham CVD risk in the whole cohort.

Predictors		*n*	OR (95% CI)			
			Model 1	Model 2	Model 3	Model 4
OSA	Control	823	References	References	References	References
	Mild	686	*1.919 (1.469–2.508)	*1.818 (1.389–2.380)	*1.555 (1.178–2.053)	*1.551 (1.172–2.052)
	Moderate	742	*3.955 (3.083–5.073)	*3.600 (2.797–4.634)	*3.014 (2.324–3.908)	*3.041 (2.338–3.955)
	Severe	2224	*5.137 (4.136–6.380)	*4.346 (3.457–5.465)	*3.472 (2.743–4.394)	*3.365 (2.626–4.310)
	*P* for linear trend		*P* < 0.001	*P* < 0.001	*P* < 0.001	*P* < 0.001
SWS%	≥20.1	1123	References	References	References	References
	12.8–20.1	1124	*1.295 (1.075–1.559)	*1.258 (1.043–1.519)	1.197 (0.983–1.458)	*1.243 (1.019–1.516)
	5.9–12.8	1114	*1.760 (1.467–2.111)	*1.649 (1.371–1.983)	*1.601 (1.320–1.941)	*1.597 (1.315–1.940)
	<5.9	1114	*2.561 (2.140–3.064)	*2.377 (1.982–2.851)	*2.281 (1.886–2.759)	*2.139 (1.763–2.595)
	*P* for linear trend		*P* < 0.001	*P* < 0.001	*P* < 0.001	*P* < 0.001
EDS	≤10	2945	References	References	References	References
	>10	1530	*1.770 (1.557–2.012)	*1.574 (1.380–1.795)	*1.427 (1.243–1.639)	*1.331 (1.153–1.536)
Sleep duration	≥8 h	455	1.106 (0.891–1.374)	1.081 (0.867–1.347)	1.126 (0.894–1.419)	1.161 (0.919–1.467)
	7–8 h	1518	References	References	References	References
	6–7 h	1355	0.872 (0.746–1.018)	0.897 (0.766–1.050)	0.952 (0.807–1.123)	0.934 (0.789–1.106)
	5–6 h	681	0.926 (0.795–1.163)	1.021 (0.842–1.239)	1.062 (0.867–1.300)	1.032 (0.830–1.283)
	<5 h	466	1.007 (0.811–1.251)	1.122 (0.900–1.400)	1.239 (0.983–1.563)	1.085 (0.818–1.438)

Model 1 was a univariate logistic analysis, and Model 2 was a multivariate analysis after adjusting sex, BMI and alcohol use, and Model 3 was after adjusting sex, BMI, alcohol use, fasting glucose, LDL-c, and hyperlipidemia, while Model 4 increased the adjustment of sleep variables (MAI, SE, and TST/ESS/SWS/AHI) on the basis of Model 3. OR, odds ratio; CI, confidence interval; OSA, obstructive sleep apnea; SWS, slow-wave sleep; EDS, excessive sleep sleepiness; SSD, short sleep duration; CVD, cardiovascular diseases; BMI, body mass index; LDL-C, low-density lipoprotein; MAI, microarousal index; SE: sleep efficiency; TST, total sleep time; ESS, Epworth Sleepiness Scale; AHI, apnea hypopnea index.

*P indicates a significant difference.

Both low SWS and EDS are common in subjects with OSA; we thus explored the impact of low SWS and EDS on the 10-year CVD risk depended on the presence of OSA. We respectively investigated the impact of low SWS and EDS on the CVD risk in participants with and without OSA (Table 4). The adjustment models were those of Table 3. We found that low SWS (<12.8%) increased the 10-year risk of CVD whether in the population with OSA or controls. Compared to the first quartile (SWS ≥ 20.1%), the association between the 10-year risk of CVD and SWS became stronger (In OSA cohort: OR = 1.197, 95% CI: 0.968–1.480; OR = 1.524, 95% CI: 1.239–1.874; OR = 2.121, 95% CI: 1.729–2.603; In control cohort: OR = 1.448, 95% CI: 0.778–2.693; OR = 2.153, 95% CI: 1.155–4.015; OR = 2.200, 95% CI: 1.140–4.247) as SWS decreased in Model 4. In subjects with OSA, those with EDS were more likely to suffer from CVD than those lacking EDS, but no impact of EDS was evident in the control group. Consistent with data from the whole cohort, sleep duration was not significantly associated with an increased 10-year risk of CVD.

**TABLE 4:** Association of SWS, EDS, SSD and moderate-to-high Framingham CVD risk in stratified analysis.

Predictors			*n*	OR (95% CI)			
				Model 1	Model 2	Model 3	Model 4
OSA	SWS%	≥20.1	842	References	References	References	References
		12.8–20.1	889	*1.246 (1.018–1.524)	*1.239 (1.012–1.517)	1.169 (0.946–1.445)	1.197 (0.968–1.480)
		5.9–12.8	941	*1.568 (1.288–1.909)	*1.537 (1.261–1.873)	*1.520 (1.238–1.866)	*1.524 (1.239–1.874)
		<5.9	980	*2.268 (1.870–2.752)	*2.206 (1.817–2.680)	*2.152 (1.758–2.634)	*2.121 (1.729–2.603)
		*P* for linear trend		*P* < 0.001	*P* < 0.001	*P* < 0.001	*P* < 0.001
	EDS	≤10	2265	Reference	References	References	References
		>10	1387	*1.577 (1.376–1.807)	*1.500 (1.306–1.723)	*1.384 (1.198–1.598)	*1.359 (1.171–1.577)
	Sleep duration	≥8 h	397	1.089 (0.866–1.370)	1.072 (0.851–1.350)	1.111 (0.874–1.413)	1.168 (0.916–1.490)
		7–8 h	1282	References	References	References	References
		6–7 h	1098	0.936 (0.793–1.105)	0.944 (0.799–1.115)	0.998 (0.839–1.188)	0.981 (0.821–1.171)
		5–6 h	536	1.039 (0.846–1.277)	1.066 (0.867–1.312)	1.125 (0.906–1.396)	1.106 (0.877–1.394)
		<5 h	339	1.127 (0.884–1.438)	1.190 (0.931–1.521)	*1.297 (1.004–1.674)	1.185 (0.874–1.606)
Control	SWS%	≥20.1	281	References	References	References	References
		12.8–20.1	235	1.273 (0.721–2.249)	1.177 (0.659–2.100)	1.312 (0.716–2.405)	1.448 (0.778–2.693)
		5.9–12.8	173	*2.141 (1.223–3.749)	*1.962 (1.105–3.485)	*1.942 (1.058–3.565)	*2.153 (1.155–4.015)
		<5.9	134	*2.475 (1.380–4.438)	*2.532 (1.390–4.610)	*2.271 (1.193–4.325)	*2.200 (1.140–4.247)
	EDS	≤10	680	References	References	References	References
		>10	143	1.207 (0.728–2.003)	1.070 (0.638–1.797)	0.956 (0.549–1.662)	1.015 (0.576–1.789)
	Sleep duration	≥8 h	58	0.919 (0.401–2.103)	0.982 (0.421–2.290)	1.023 (0.415–2.518)	1.031 (0.418–2.542)
		7–8 h	236	References	References	References	References
		6–7 h	257	0.619 (0.358–1.069)	0.668 (0.382–1.167)	0.705 (0.392–1.269)	0.634 (0.348–1.157)
		5–6 h	145	0.919 (0.508–1663)	1.008 (0.549–1.850)	0.793 (0.409–1.535)	0.564 (0.277–1.146)
		<5 h	127	1.270 (0.713–2.261)	1.339 (0.741–2.422)	1.378 (0.734–2.588)	0.559 (0.234–1.336)

Model 1 was a univariate logistic analysis, and Model 2 was a multivariate analysis after adjusting sex, BMI and alcohol use, and Model 3 was after adjusting sex, BMI, alcohol use, fasting glucose, LDL-c, and hyperlipidemia, while Model 4 increased the adjustment of sleep variables (MAI, SE, and TST/ESS/SWS/AHI) on the basis of Model 3. OR, odds ratio; CI, confidence interval; OSA, obstructive sleep apnea; SWS, slow-wave sleep; EDS, excessive sleep sleepiness; SSD, short sleep duration; CVD, cardiovascular diseases; BMI, body mass index; LDL-C, low-density lipoprotein; MAI, microarousal index; SE: sleep efficiency; TST, total sleep time; ESS, Epworth Sleepiness Scale; AHI, apnea hypopnea index.

*P indicates a significant difference.

In stratified analysis by OSA severity, logistic regression was also used to evaluate the association between the 10-year risk of CVD and SWS, EDS, and sleep duration (Table 5). Models 1–4 were as described above. In the mild-to-moderate OSA group, compared with those with SWS ≥ 20.1, only SWS < 5.9% was significantly associated with an increased 10-year CVD risk after adjusting for metabolic and sleep parameters. Unfortunately, subjective EDS and sleep duration did not show any association with CVD risk in patients with mild-to-moderate OSA. In the severe OSA group, the impact of SWS on the 10-year CVD risk was extremely significant. The risk was increased with any SWS duration, and the less the SWS the higher the CVD risk (OR = 1.410, 95% CI: 1.059–1.878; OR = 1.552, 95% CI: 1.178–2.044; OR = 2.214, 95% CI: 1.700–2.882). In addition, subjective EDS had a significant effect on the increased 10-year risk of CVD only in severe OSA group. Nevertheless, In the population with OSA, no obvious association between sleep duration and the CVD risk was apparent.

**TABLE 5:** Association of SWS, EDS, SSD and moderate-to-high Framingham risk in stratified analysis by OSA severity.

Predictors			*n*	OR (95% CI)			
				Model 1	Model 2	Model 3	Model 4
Mild OSA	SWS%	≥20.1	202	References	References	References	References
		12.8–20.1	202	0.717 (0.431–1.192)	0.685 (0.725–1.672)	0.605 (0.353–1.037)	0.618 (0.359–1.063)
		5.9–12.8	157	1.483 (0.912–2.413)	1.462 (0.890–2.401)	1.388 (0.830–2.320)	1.436 (0.852–2.421)
		<5.9	125	*1.793 (1.080–2.975)	*2.048 (1.218–3.443)	*2.095 (1.226–3.582)	*2.114 (1.234–3.620)
	EDS	≤10	520	References	References	References	References
		>10	166	1.180 (0.786–1.771)	1.154 (0.766–1.739)	1.131 (0.742–1.725)	1.111 (0.725–1.704)
	Sleep duration	≥8 h	45	0.645 (0.271–1.535)	0.636 (0.266–1.525)	0.640 (0.261–1.568)	0.640 (0.259–1.582)
		7–8 h	216	References	References	References	References
		6–7 h	214	0.906 (0.571–1.437)	0.914 (0.574–1.455)	0.913 (0.564–1.477)	0.940 (0.576–1.534)
		5–6 h	126	1.293 (0.779–2.149)	1.275 (0.765–2.127)	1.283 (0.757–2.174)	1.366 (0.773–2.415)
		<5 h	85	1.458 (0.828–2.569)	1.419 (0.802–2.513)	1.319 (0.724–2.402)	1.476 (0.715–3.049)
Moderate OSA	SWS%	≥20.1	203	References	References	References	References
		12.8–20.1	206	1.248 (0.826–1.887)	1.239 (0.818–1.876)	1.191 (0.774–1.831)	1.181 (0.766–1.822)
		5.9–12.8	207	*1.553 (1.033–2.335)	1.500 (0.994–2.261)	1.497 (0.980–2.286)	1.503 (0.981–2.301)
		<5.9	126	*2.348 (1.483–3.717)	*2.376 (1.498–3.769)	*2.508 (1.557–4.039)	*2.500 (1.543–4.050)
	EDS	≤10	551	References	References	References	References
		>10	191	1.098 (0.784–1.539)	1.096 (0.781–1.537)	1.046 (0.736–1.487)	1.005 (0.703–1.437)
	Sleep duration	≥8 h	66	0.856 (0.481–1.523)	0.882 (0.495–1.572)	0.946 (0.522–1.715)	0.902 (0.491–1.657)
		7–8 h	236	References	References	References	References
		6–7 h	223	0.996 (0.681–1.456)	1.015 (0.693–1.486)	1.017 (0.685–1.510)	1.017 (0.679–1.523)
		5–6 h	134	1.230 (0.797–1.897)	1.258 (0.814–1.944)	1.162 (0.739–1.829)	1.243 (0.759–2.037)
		<5 h	83	1.312 (0.789–2.181)	1.345 (0.806–2.243)	1.462 (0.863–2.474)	1.514 (0.765–2.996)
Severe OSA	SWS%	≥20.1	437	References	References	References	References
		12.8–20.1	481	*1.433 (1.097–1.873)	*1.422 (1.087–1.860)	*1.402 (1.058–1.859)	*1.410 (1.059–1.878)
		5.9–12.8	577	*1.484 (1.147–1.918)	*1.489 (1.150–1.927)	*1.504 (1.149–1.969)	*1.552 (1.178–2.044)
		<5.9	729	*2.095 (1.639–2.677)	*2.086 (1.630–2.668)	*2.068 (1.598–2.677)	*2.214 (1.7–2.882)
	EDS	≤10	1194	References	References	References	References
		>10	1030	*1.565 (1.322–1.852)	*1.560 (1.315–1.850)	*1.436 (1.202–1.716)	*1.597 (1.327–1.922)
	Sleep duration	≥8 h	286	1.123 (0.858–1.471)	1.125 (0.858–1.474)	1.168 (0.880–1.551)	1.295 (0.969–1.731)
		7–8 h	830	References	References	References	References
		6–7 h	661	0.966 (0.786–1.187)	0.974 (0.792–1.198)	1.051 (0.847–1.306)	1.026 (0.821–1.283)
		5–6 h	276	1.039 (0.790–1.367)	1.062 (0.807–1.398)	1.196 (0.898–1.593)	1.121 (0.823–1.527)
		<5 h	171	1.147 (0.824–1.595)	1.201 (0.861–1.674)	1.308 (0.925–1.848)	1.038 (0.691–1.559)

Model 1 was a univariate logistic analysis, Model 2 was a multivariate analysis after adjusting sex, BMI and alcohol use, and Model 3 was after adjusting sex, BMI, alcohol use, fasting glucose, LDL-c, and hyperlipidemia, while Model 4 increased the adjustment of sleep variables (AHI, MAI, SE, and TST/ESS/SWS) on the basis of Model 3. OR, odds ratio; CI, confidence interval; OSA, obstructive sleep apnea; SWS, slow-wave sleep; EDS, excessive sleep sleepiness; SSD, short sleep duration; CVD, cardiovascular diseases; BMI, body mass index; LDL-C, low-density lipoprotein; MAI, microarousal index; SE, sleep efficiency; TST, total sleep time; ESS, Epworth Sleepiness Scale.

*P indicates a significant difference.

## Discussion

With the increase of the understanding of sleep structure, the effects of SWS, EDS and sleep duration have attracted great interest. We explored the effects of OSA, low SWS, EDS, and SSD on the 10-year risk of CVD. We found that: (1) Patients with OSA were at a significantly increased 10-year risk of CVD compared to controls; as OSA severity increased, the risk of CVD increased significantly; (2) whether in patients with OSA or controls, low SWS (<12.8%) would increase the risk of CVD. In the mild-to-moderate OSA group, only low SWS (<5.9%) significantly increased the risk of CVD, whereas in the severe OSA group, the CVD risk was increased with any SWS duration; (3) the relationship between subjective EDS and CVD risk only existed in severe OSA group; and, (4) the impact of sleep duration on CVD risk might be minor regardless of the presence of OSA.

Obstructive sleep apnea is one of the most common sleep-disordered breathing diseases; OSA is multifactorial, being associated with all of sleep fragmentation, intermittent hypoxia, sympathetic activation, and oxidative stress; all contribute to CVD, which is very common in patients with OSA. Several observational studies found that untreated OSA significantly impacted CVD morbidity and mortality (3). We here reconfirm the effect of OSA on the CVD risk.

We found that SWS in OSA patients was indeed lower than in those without OSA. Low SWS (<12.8%) was a key factor for CVD risk in both OSA group and control group. In the mild-to-moderate OSA group, patients with extremely low SWS (SWS < 5.9%) were more likely to suffer from CVD compared with those with higher SWS (SWS ≥ 20.1). In those with severe OSA, all SWS-decreased subgroups were at higher risks of CVD; the OR increased as SWS decreased. Based on our results, the role of low SWS in mild-to-moderate OSA seemed to have been reduced, and the reasons were not clear. SWS is thought to be linked to hormone secretion, immunoregulation and metabolic homeostasis (32–34). We previously found that OSA patients with low SWS were more susceptible to insulin resistance (30). SWS inhibition in young healthy adults could even reduce insulin-sensitivity, impair glucose tolerance, and increase the risk of diabetes (33). Similarly, we found that low SWS (<12.8%) indeed increased the risk of CVD in healthy subjects. Thus, low SWS requires treatment, regardless of the presence of OSA. And it is necessary to extend PSG to more people and find treatment which can effectively improve the SWS content of patients. However, the SWS proportion decreases with age; the reason remains unclear. Neuronal loss or reduced synaptic strength may be in play (35). Aging also greatly impacts the FRS. Therefore, although the influence of SWS on CVD cannot be ignored, the precise means by which low SWS affects the CVD risk requires further research.

The relationship between ESS score and the severity of OSA is still unclear. Several studies have investigated that EDS may be attributable to many factors, not merely hypoxia events and frequent arousals caused by sleep fragmentation (36, 37). Because of the insufficient understanding of EDS and the ambiguous description reported by patients, so it is difficult to evaluate the exact role of EDS in the diseases. EDS is associated with obesity and hypertension, independently of OSA (38, 39). However, it has been argued that EDS is a significant predictor of OSA-related CVD (40). We found that EDS was not associated with an increased CVD risk in the control population. Only in those with severe OSA, patients with EDS were more likely to develop CVD than those without EDS. Therefore, the impact of EDS on an increased risk of CVD is highly dependent on OSA severity; the precise role played by EDS remains unclear. Further prospective, randomized controlled studies are required.

We previously showed that patients with OSA were more likely to exhibit IR when the sleep duration was <5 h (41). A meta-analysis reported increased CVD mortality at sleep durations ≤ 5 and ≥ 9 h (42). However, we found that any effect of sleep duration on CVD risk was not significant in either the whole cohort or the OSA cohort. According to our existing data, the role of sleep duration could not be judged, but it was impossible to eliminate completely any role for sleep duration. Many patients reported that the sleep duration monitored by standard PSG would be changed due to uncomfortableness (e.g., many electrodes on the body and inability to turn over). We speculated that the sleep duration monitored by PSG could not reflect the daily sleep duration due to individual sleep habits, thus concealing the role of sleep duration in CVD risk. Another important consideration in the casual relationship between sleep duration and CVD is also unsolved. Up to now, the studies on sleep duration are mostly cross-sectional research, so it is impossible to define the causal relationship between sleep duration and CVD. Whether the change of sleep duration promotes the pathogenesis of CVD or the sleep duration is affected after the pathogenesis of CVD. It is even possible that positive feedback is in play: a certain sleep duration promotes CVD pathogenesis; CVD then affects sleep duration. An understanding of the precise relationship between sleep duration and CVD requires a large prospective study.

In short, our study was based on large samples, and the data were comprehensive and reliable. The diagnostic criteria of OSA were in strict accordance with the gold standard-PSG. Secondly, the sample control was strict. We excluded the samples with CVD before enrolled in the study to avoid the interference of other factors. In addition, we comprehensively studied the relationship between common sleep disturbances and the moderate-to-high risk of CVD in patients with OSA and control participants, rather than focus on the role of a single factor in the overall population, which might mask its own contribution. At the same time, our work also had certain limitations. First, there might be deviations in the evaluation of some indicators. EDS was judged by questionnaire and sleep duration was easily affected due to the PSG environment and the subject’s mental state, so it might have a certain impact on the results due to the individual difference. Second, as our study was cross-sectional in nature, we cannot address causality. Therefore, the contribution of this study to intervention was limited. Finally, FRS itself suffered from several major drawbacks. In addition to the above high-risk factors, CVD had many other high-risk factors, such as family history and diet habits, which were not included in the calculation method of FRS. The FRS overemphasized age, and subjects in our moderate-to-high risk group were indeed older. Importantly, Sleep parameters also changed with age. Therefore, future research might need the improved evaluation model of CVD risk.

## Conclusion

Low SWS would increase the 10-year risk of CVD in whether control participants and patients with OSA. And subjective EDS played a marked effect merely in patients with severe OSA. However, any effect of sleep duration on CVD was not significant in either the whole cohort or the OSA group. And the role of EDS and low SWS in 10-year risk of CVD still need to elaborated in future studies. Patients exhibiting low SWS should take note of CVD concern regardless of the presence of OSA. If patients with severe OSA are accompanied by obvious EDS, they should pay more attention to regularly conduct cardiovascular examination and take measures in advance to prevent CVD.

## Data availability statement

The raw data supporting the conclusions of this article will be made available by the authors, without undue reservation.

## Ethics statement

The studies involving human participants were reviewed and approved by Ethics Committee of Shanghai Jiao Tong University Affiliated Sixth People’s Hospital (trial registration no: ChiCTr1900025714). The patients/participants provided their written informed consent to participate in this study.

## Author contributions

XL, DQ, and HX had full access to all of the data in the study and took responsibility for the integrity of the data and the accuracy of the data analysis. HX, DQ, and SY designed the study. XZ, WH, XL, KW, FW, CL, YL, JZ, HZ, JG, and HY collected the data. XZ and WH contributed to the statistical analysis and drafted the manuscript. All authors take responsibility and vouch for the accuracy and completeness of the data and analyses and approved the manuscript.

## Abbreviations

OSA: obstructive sleep apnea
CVD: cardiovascular disease
SWS: low slow-wave sleep
EDS: excessive daytime sleepiness
SSD: short sleep duration
PSG: polysomnography
FRS: Framingham risk score
EEG: electroencephalogram
NREM: non-rapid eye movement
REM: rapid eye movement
ESS: Epworth sleepiness scale
CPAP: continuous positive airway pressure
AASM: American Academy of Sleep Medicine
ECG: electrocardiogram
EOG: electrooculogram
WC: waist circumference
HC: hip circumference
NC: neck circumference
SBP: systolic blood pressure
DBP: diastolic blood pressure
TC: total cholesterol
HDL-c: high-density-lipoprotein C
LDL-c: low-density-lipoprotein C
BMI: body mass index
WHR: waist-to-hip ratio
HOMA-IR: homeostasis model assessment of insulin resistance
AHI: apnea hypopnea index
TST: total sleep time
SE: sleep efficiency
LSpO2: lowest oxygen saturation
ODI: oxygen desaturation index
MAI: microarousal index
OR: odds ratios
CI: confidence intervals.
