# Supplementary material for: Associations between common sleep disturbances and cardiovascular risk in patients with obstructive sleep apnea: A large-scale cross-sectional study
Source: Front Cardiovasc Med. 2022 Oct 31;9:1034785. doi: 10.3389/fcvm.2022.1034785 (PMC9659611; doi:10.3389/fcvm.2022.1034785)
Supplement: Supplementary file 1 [file Data_Sheet_1.docx]

**Supplementary Material**

**Table S1** Multinomial logistic regression analysis of factors associated with Framingham CVD risk in overall participants.

| **Characteristics** |  | **FRS** | | |
| --- | --- | --- | --- | --- |
|  |  | **Low (< 10%)** | **Intermediate (10–20%)** | **High (> 20%)** |
| **BMI**≥**25** | OR | Ref. | 0.956 | 0.813 |
|  | 95% CI | 1 | 0.817-1.119 | *0.684-0.966 |
| **TG** | OR | Ref. | 1.175 | 1.173 |
|  | 95% CI | 1 | *1.122-1.230 | *1.116-1.232 |
| **LDL-c** | OR | Ref. | 1.236 | 1.254 |
|  | 95% CI | 1 | *1.131-1.352 | *1.136-1.384 |
| **HOMA-IR>2.7** | OR | Ref. | 1.065 | 1.098 |
|  | 95% CI | 1 | *1.037-1.093 | *1.070-1.127 |
| **Hyperlipidemia** | OR | Ref. | 2.831 | 4.073 |
|  | 95% CI | 1 | *2.359-3.398 | *3.362-4.936 |
| **OSA** | OR | Ref. | 4.008 | 4.298 |
|  | 95% CI | 1 | *3.071-5.231 | *3.148-5.967 |
| **TST** | OR | Ref. | 0.999 | 1.000 |
|  | 95% CI | 1 | 0.999-1.000 | 1.000-1.002 |
| **ESS>10** | OR | Ref. | 1.758 | 1.786 |
|  | 95% CI | 1 | *1.505-2.053 | *1.502-2.123 |
| **SWS** | OR | Ref. | 0.978 | 0.955 |
|  | 95% CI | 1 | *0.971-0.985 | *0.946-0.964 |

CVD, cardiovascular diseases; OR, odds ratio; CI, confidence interval; BMI, body mass index; TG, triglyceride; LDL-C, low-density lipoprotein; HOMA-IR, homeostasis model assessment for insulin resistance; OSA, obstructive sleep apnea; TST, total sleep time; ESS, epworth sleepiness scale; SWS, slow-wave sleep. * p indicates a significant difference.

**Figure S1** Flowchart of participants enrollment.


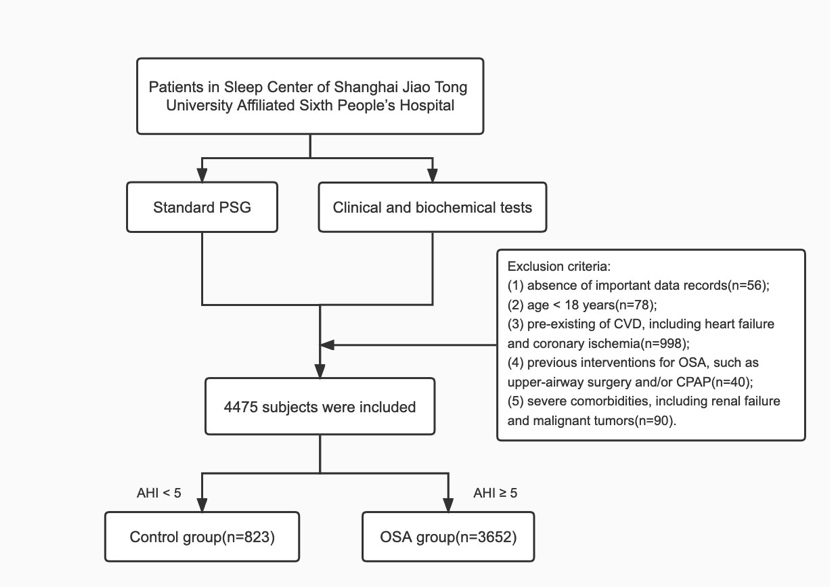


AHI, apnea hypopnea index; OSA, obstructive sleep apnea; CVD: cardiovascular disease; CPAP: continuous positive airway pressure. A total of 56 patients were not included in the study because of missing data. Specifically, there were some problems with the sleep monitoring data of these patients. Because patients could not adapt to the testing equipment or fell off midway, the sleep duration recorded by sleep monitoring was insufficient (usually less than 4 hours), and these patients refused to conduct PSG monitoring again, so these highly variable records could not support data analysis.

*Sample size calculation*

All subjects in this study went to the sleep center of Shanghai Jiao Tong University Affiliated Sixth People’s Hospital for medical help due to snoring. The sample size estimation was determined based on the previous study as described ^1^.

Taking SWS as an example, the formulas were as followed:

$\mu_{A}=15.15$ (mean of Group A)

$\mu_{B}=11.76$ (mean of Group B)

$\kappa=\frac{n_{A}}{n_{B}}=1$ (sampling ratio)

$\sigma=10.77$ (standard deviation)

$$n_{B}=\left( 1+\frac{1}{\kappa} \right)\times\left( \sigma\times\frac{Z_{1-\frac{\alpha}{2}}+Z_{1-\beta}}{\mu_{A}-\mu_{B}} \right)^{2}$$

$$1-\beta=\phi\left( Z-Z_{1-\frac{\alpha}{2}} \right)+\phi\left( -Z-Z_{1-\frac{\alpha}{2}} \right)$$

$$Z=\frac{\mu_{A}-\mu_{B}}{\sigma\times\sqrt{\frac{1}{n_{A}}+\frac{1}{n_{B}}}}$$

We estimated that a power of 90% could be achieved at a significance level of 5% with 212 subjects.

Taking EDS as an example, the formulas were as followed:

$$n_{B}=\left( p_{B}(1-p_{B})+\frac{p_{A}(1-p_{A})}{\kappa} \right)\times\left( \frac{Z_{1-\frac{\alpha}{2}}+Z_{1-\beta}}{p_{A}-p_{B}} \right)^{2}$$

$$1-\beta=\phi\left( Z-Z_{1-\frac{\alpha}{2}} \right)+\phi\left( -Z-Z_{1-\frac{\alpha}{2}} \right)$$

$$Z=\frac{p_{A}-p_{B}}{\sqrt{\frac{p_{A}(1-p_{A})}{n_{A}}+\frac{p_{B}(1-p_{B})}{n_{B}}}}$$

We estimated that a power of 90% could be achieved at a significance level of 5% with 278 subjects.

Our sample size was far more than the minimum sample size required.

Reference:

1. Wang X, Ji X. Sample Size Estimation in Clinical Research: From Randomized Controlled Trials to Observational Studies. Chest. 2020; 158(1s): S12-s20.
